# Interplay Between Higher-Order Information Diffusion and Epidemic Spreading Under Asymmetric Intercity Commuting

**DOI:** 10.3390/e28101163

**Published:** 2026-10-21

**Authors:** Zhishuang Wang, Yingjie Liang, Huiyu Zhou, Qian Yin, Shaoyong Li, Zhiyong Hong

**Affiliations:** School of Electronics and Information Engineering, Wuyi University, Jiangmen 529020, China; zswang@wyu.edu.cn (Z.W.);

**Keywords:** coupled information–epidemic spreading, multilayer networks, higher-order interactions, intercity commuting

## Abstract

Information dissemination and intercity commuting jointly shape epidemic dynamics, but their combined effects in heterogeneous cities require further investigation. We develop a two-layer model coupling information diffusion through pairwise interactions and 2-simplices with susceptible–infected–susceptible disease transmission in two cities differing in population size and transmission conditions. Asymmetric commuting generates additional cross-city contacts while preserving within-city contact structures, and informed individuals receive protection against infection. Using the microscopic Markov chain method, we derive the critical transmission probability $\beta_{c}$ from the spectral radius of an effective transmission matrix. The predicted stationary infection densities agree well with Monte Carlo simulations. Stronger pairwise information transmission suppresses infection and raises $\beta_{c}$, with the latter effect approaching saturation. Under the investigated conditions, higher-order information transmission mainly changes the distribution of information states and has a comparatively limited effect on prevalence. Stronger commuting generally increases prevalence, whereas its influence on $\beta_{c}$ depends on the city network structure. Variations in information-induced protection produce larger threshold changes than variations in commuting intensity over the examined parameter ranges. Moreover, faster withdrawal from active information dissemination weakens epidemic suppression even when individual protection persists. These findings clarify the distinct roles of sustained information dissemination, individual protection, and commuting contacts in coupled epidemic dynamics.

## 1. Introduction

The widespread transmission of infectious diseases is not determined solely by biological contagion but is also strongly influenced by human awareness, behavioral responses, and the circulation of disease-related information [1,2,3]. During an epidemic, individuals may acquire information from social contacts, online platforms, or public communication channels and subsequently adopt protective behaviors, such as reducing unnecessary contacts or taking personal preventive measures [4]. At the same time, the progression of an epidemic can stimulate information acquisition and dissemination, forming a bidirectional interaction between information diffusion and disease spreading [5,6]. Such feedback may substantially alter the prevalence, outbreak threshold, and long-term evolution of an epidemic [7]. Consequently, understanding the coupled dynamics of information and epidemic spreading has become an important issue in network epidemiology and provides a useful framework for revealing how individual behavioral responses reshape collective epidemic dynamics.

Complex-network approaches provide a natural framework for describing the interactions underlying coupled information–epidemic spreading [8,9]. In realistic systems, however, the two spreading processes generally operate through different interaction structures. Information can spread over long distances through online communication and social relationships and may involve not only pairwise interactions but also group-level reinforcement [10,11,12], whereas disease transmission relies primarily on physical contacts that are more strongly constrained by spatial locations [13]. Higher-order networks explicitly represent group interactions and provide a framework for investigating how these interactions shape collective dynamics [14,15,16]. For contagion processes on these networks, group structure and nonlinear transmission mechanisms can alter spreading thresholds and the nature of phase transitions [17,18]. Moreover, population movement between cities can generate additional physical contacts and connect otherwise separated epidemic processes [19,20,21]. In particular, commuting between cities is typically temporary rather than permanent: individuals retain their routine contacts in the resident city while creating additional contacts in the destination city before returning [22,23]. These characteristics suggest that a realistic description of coupled information–epidemic dynamics should simultaneously account for multilayer network structures, higher-order information interactions, and cross-city commuting, especially when cities differ in population size and local transmission environments.

Studies of coupled information–epidemic dynamics have extended multilayer models to incorporate higher-order interactions and heterogeneous behavioral responses. Recent studies have shown that the effects of higher-order interactions depend on their microscopic organization, including the overlap between interactions of different orders and among hyperedges of the same order [24,25]. Adaptive group membership and non-Markovian spreading processes provide further extensions for describing higher-order contagion dynamics [26,27]. Sun et al. [28] incorporated accumulated risk perception and individual heterogeneity into a simplicial-complex framework, examining their joint effects with higher-order interactions on information and epidemic spreading. Bao and Wu [29] investigated competing positive and negative preventive information on higher-order networks, revealing how such competition influences epidemic spreading through vaccination decisions. Song et al. [30] combined higher-order infection with degree-biased information diffusion, finding that information dissemination without degree preference provided more effective epidemic suppression under their model settings. To account for spatial spreading, further studies have extended information–epidemic models to metapopulation networks. An et al. [31] introduced a stranding mechanism determined by infection levels and medical resources to describe changes in patch population sizes during individual mobility. Chen and Huo [32] distinguished intra-patch and inter-patch information contacts in a higher-order metapopulation model, showing that stronger inter-patch information interactions can suppress epidemic spreading. Li et al. [33] introduced awareness-based event-triggered migration into a network of residences and transfer stations, demonstrating that adaptive mobility can reduce infection peaks and epidemic size.

Research on commuting-driven epidemic dynamics has further examined model formulation, spatial heterogeneity, and intervention strategies. Foundational studies have established analytical frameworks linking recurrent mobility and multilayer mobility structures to epidemic thresholds and critical spreading regimes [34,35]. Regarding model formulation, Bliman et al. [36] represented commuting through the periodic mixing and separation of subpopulations, establishing a framework for analyzing the basic reproduction number and the stability of disease-free equilibria. Li et al. [37] distinguished contact mechanisms at homes and destinations in a metapopulation model with recurrent mobility, revealing a nonmonotonic relationship between mobility probability and the epidemic threshold. Regarding spatial heterogeneity, Cho et al. [38] assigned commuting populations to network edges and incorporated mobile telecommunication data to capture regional differences in infection patterns and peak timing. Chen et al. [39] considered multiple transportation modes together with random and regular commuting, showing that regular commuting can have opposing effects on epidemic size and amplify regional disparities in spreading. Data-driven studies further show that heterogeneous mobility and contact patterns can help explain unequal epidemic exposure [40,41]. Comparisons of mobility-derived and survey-based contact matrices provide additional evidence for incorporating time-varying behavior into epidemic models [42]. Meanwhile, the sensitivity of inferred mobility patterns to data-processing choices highlights the need for careful interpretation of empirical mobility networks [43]. Regarding interventions, Zeng et al. [44] developed a multilayer commuting model using transportation and epidemic data from Shanghai to evaluate mobility restrictions, personal protection, and testing efficiency. Alrawas et al. [45] combined directional intercity mobility with age structure and vaccination, finding that the required mobility restrictions depend on population characteristics and vaccination coverage, and that restrictions targeting selected cities can outperform uniform reductions across the network. Mobility-targeted interventions have also been shown to depend on the underlying transmission mechanism, reinforcing the importance of specifying how movement generates infectious contacts [46]. These developments motivate further investigation of the joint effects of higher-order information diffusion and asymmetric intercity commuting, particularly when cities differ in population size and transmission conditions, and routine within-city contacts coexist with additional cross-city contacts.

Although previous studies have coupled information diffusion with population mobility, further attention is needed to distinguish commuting-induced cross-city contacts from persistent within-city contact structures and to clarify how they interact with higher-order information spreading. When cities differ in population size and transmission conditions, additional contacts generated by commuting may alter infection levels in each city, while information-induced protection can modulate these effects. Moreover, awareness and active information dissemination are not equivalent: individuals who stop spreading information may remain aware and maintain protective behaviors. The level of active information spreading alone may therefore be insufficient to characterize the overall suppression of disease transmission. These considerations call for a joint examination of information-state evolution, protective effects, and heterogeneous city contact structures under asymmetric intercity commuting.

To address these issues, we construct a two-layer coupled spreading model incorporating higher-order information interactions and asymmetric intercity commuting. The upper layer is a unified information network covering individuals in both cities, where information spreads through pairwise interactions and 2-simplices, with individuals distinguished according to whether they are unaware, actively disseminating information, or no longer participating in its dissemination. The lower layer comprises two city-specific contact networks with different population sizes and disease transmission conditions. Residents of the smaller city may travel to the larger city, establish additional contacts, and subsequently return, while infection through these contacts can occur in either direction between commuters and destination-city residents. We use the microscopic Markov chain (MMC) method [7,47,48] to formulate the evolution equations for node-state probabilities and derive the critical transmission probability $\beta_{c}$ by linearizing the dynamics around the disease-free state. Monte Carlo (MC) simulations are then used to assess the theoretical predictions and systematically investigate how higher-order information spreading, information-state transitions, commuting intensity, protective effects, and city network structures influence stationary spreading levels and the critical transmission probability. This framework clarifies the coupled spreading mechanisms arising from the joint effects of information-induced protection and commuting contacts.

The remainder of this paper is organized as follows. Section 2 introduces the two-layer network structure, intercity commuting mechanism, and coupled spreading rules. Section 3 develops the MMC evolution equations and derives the critical transmission probability. Section 4 validates the theoretical results through numerical simulations and examines the effects of the principal parameters and network structures. Section 5 concludes the paper.

## 2. Coupled Propagation Model

**Figure 1:**
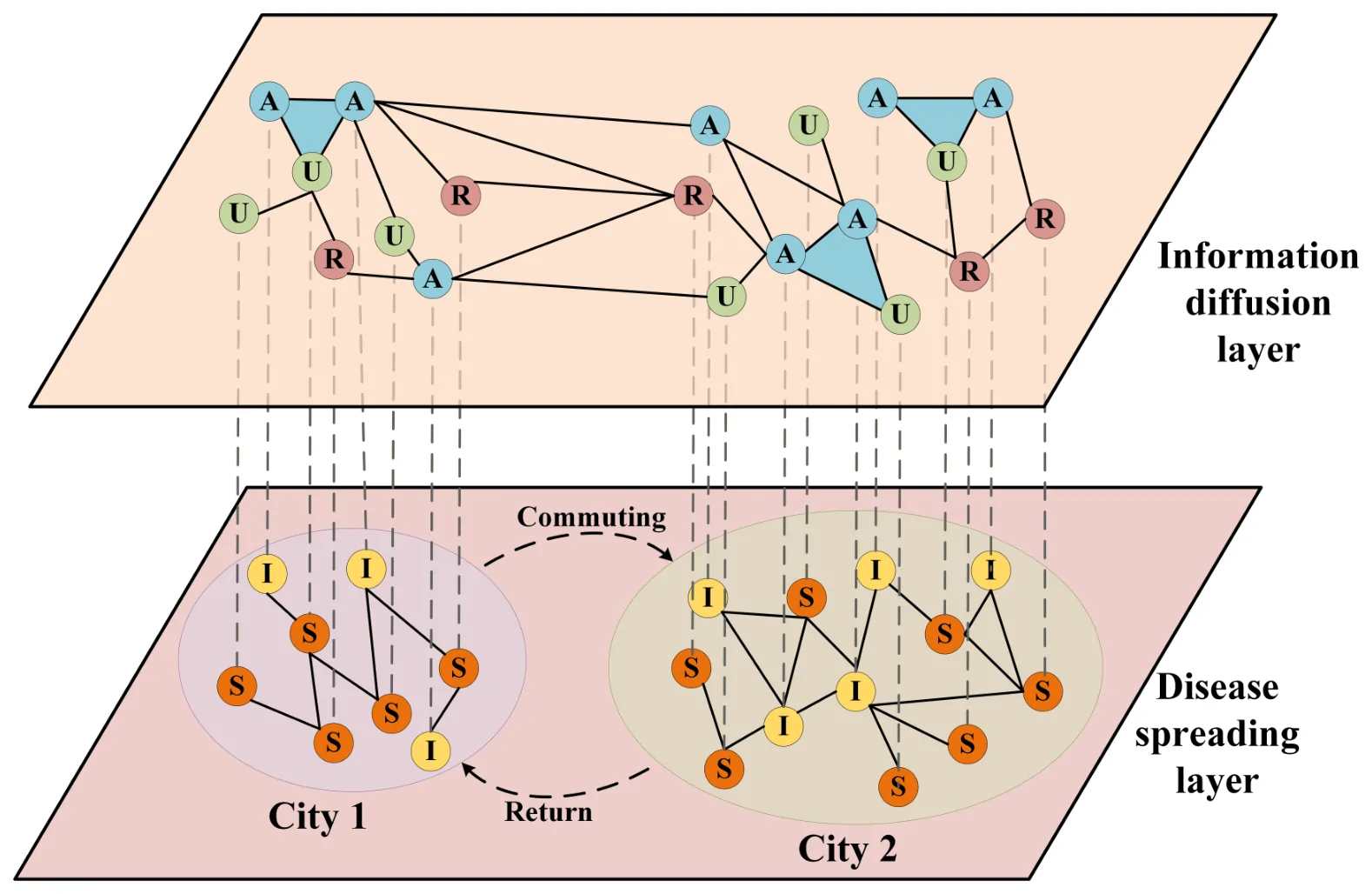
Schematic diagram of the proposed coupled information—epidemic spreading model. The upper information layer incorporates both pairwise links and 2-simplices, while the lower epidemic layer consists of two city-specific contact networks. Individuals in the smaller City 1 can commute to the larger City 2 and subsequently return, whereas reverse commuting is not considered. Dashed vertical lines denote the correspondence between nodes in the two layers.

As illustrated in Figure 1, we construct a two-layer network model composed of an information diffusion layer and a disease spreading layer. The upper layer consists of a single information diffusion network that includes individuals from both cities and characterizes information interactions among them. This unified representation is adopted because information diffusion is generally less constrained by geographical boundaries: individuals located in different cities can exchange information through online social platforms, instant communication tools, and cross-city social relationships. Therefore, individuals from the two cities can be regarded as sharing a common information environment. In addition to conventional pairwise links, 2-simplices composed of three nodes are introduced into the upper network to capture higher-order information interactions at the group level. The lower layer represents disease spreading and consists of two disease contact networks corresponding to City 1 and City 2, respectively. In contrast to information diffusion, disease transmission mainly relies on physical contacts and is therefore more spatially localized. Accordingly, the routine contact patterns within the two cities are represented by two separate networks. City 1 denotes a smaller city, whereas City 2 represents a larger city, containing $N_{1}$ and $N_{2}$ individuals, respectively, with $N_{1}<N_{2}$. The total number of individuals in the system is $N=N_{1}+N_{2}$. Each node in the upper information network corresponds to the same individual in one of the two lower-layer city networks, such that every individual simultaneously participates in the information and epidemic processes. To further account for additional disease contacts induced by population movement between the two cities, we introduce an asymmetric cross-city commuting mechanism. Specifically, individuals in City 1 commute to City 2 at a commuting probability σ, establish additional epidemic-related contacts there, and subsequently return to their resident city, whereas individuals in City 2 do not commute to City 1. A larger σ indicates a higher level of commuting from City 1 to City 2. The within-city contact networks describe relatively stable potential contact relationships among residents, whereas contact intensity reflects the effective contribution of these relationships to disease transmission during each time step. In this model, temporary commuting from City 1 to City 2 reduces the effective opportunities for contacts in the resident city and introduces contact opportunities with destination-city residents, while the original potential local contact relationships are retained. Thus, preserving within-city network structures and modifying contact intensity during commuting describe the stability of potential contact relationships and changes in effective contact opportunities, respectively, and are compatible with each other.

**Figure 2:**
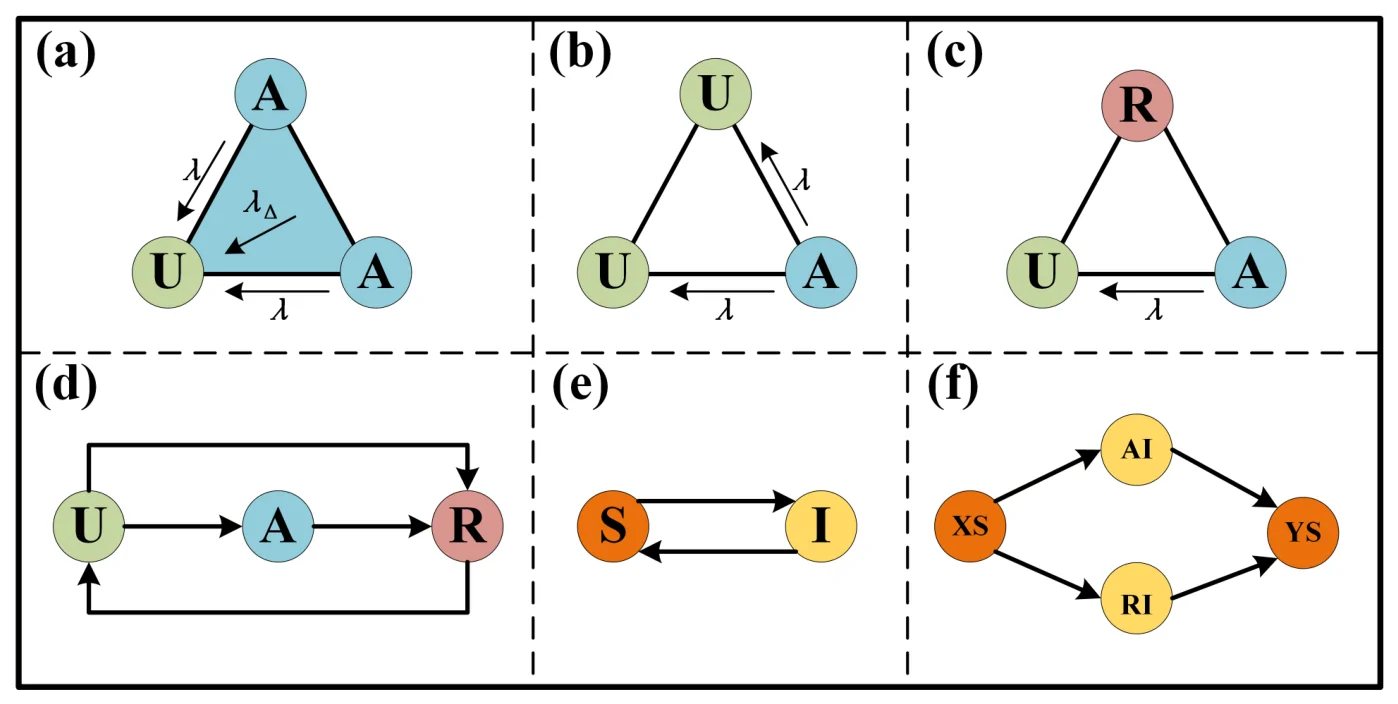
Schematic illustration of the spreading mechanisms and state transitions in the coupled information–epidemic model. (**a**–**c**) Information spreading within a 2-simplex, including pairwise and higher-order interactions. (**d**) State transitions among U, A, and R in the information diffusion layer. (**e**) State transitions between S and I in the disease spreading layer. (**f**) Coupled state transitions, where XS and YS belong to {US, AS, RS}.

In the information diffusion layer, each individual can be in one of three states: Unaware (U), Active (A), or Removed (R). An individual in state U has not yet acquired the information, whereas an individual in state A is aware of the information and actively disseminates it to neighboring individuals. An individual in state R is aware of the information but no longer participates in its further diffusion and therefore does not contribute to information transmission. To capture both ordinary interpersonal interactions and group-level reinforcement, information diffusion is modeled through pairwise links and 2-simplices. As illustrated in Figure 2a–c, through a pairwise interaction, an individual in state A transmits information to a neighboring individual in state U at probability *λ*. When only one node in a 2-simplex is in state A, the higher-order transmission mechanism is inactive, and information spreads solely through pairwise interactions. By contrast, when two nodes within the same 2-simplex are simultaneously in state A, and the remaining node is in state U, an additional higher-order transmission channel with probability $\lambda_{\Delta }$ is activated in addition to pairwise transmission. This mechanism accounts for the reinforcement effect caused by simultaneous exposure to multiple active information spreaders. Since individuals in state R do not disseminate information, they contribute neither to pairwise transmission nor to the activation of higher-order spreading. When an individual in state U acquires information through the information diffusion process, it enters state A with probability *η* and state R with probability 1−η. Thus, *η* characterizes the tendency of newly informed individuals to actively disseminate the information. As shown in Figure 2d, an individual in state A enters state R with probability *ϕ*, representing withdrawal from active information dissemination. Meanwhile, an individual in state R returns to state U with probability *δ*, reflecting the loss of awareness or memory of the information over time.

In the disease spreading layer, each individual can be in either the Susceptible (S) or Infected (I) state. A susceptible individual can become infected through two types of contacts. The first corresponds to routine contacts within the resident city, through which a susceptible individual may be infected by infected neighbors in the corresponding city-specific contact network. The second arises from additional contacts induced by cross-city commuting. For commuters from City 1, infection may occur through local contacts in City 1 or through contacts with infected individuals after traveling to City 2. Meanwhile, infected commuters from City 1 can also impose an additional infection risk on susceptible individuals in City 2. Cross-city commuting therefore provides an additional transmission channel connecting the epidemic processes in the two cities. The disease dynamics follow a susceptible–infected–susceptible (SIS) process, as illustrated in Figure 2e. Upon effective exposure, a susceptible individual changes from state S to state I, whereas an infected individual recovers with probability *μ* and returns to state S. Hence, recovered individuals do not acquire permanent immunity and remain susceptible to reinfection.

To account for differences in population size and contact environments between the two cities, $\beta_{1}$ and $\beta_{2}$ are used to characterize the disease transmission probabilities in City 1 and City 2, respectively. Moreover, acquiring disease-related information may encourage individuals to adopt protective behaviors, such as reducing social contacts or taking personal protective measures, thereby decreasing their infection risk. Let $\beta_{1}^{U}$ and $\beta_{2}^{U}$ denote the disease transmission probabilities associated with susceptible individuals in state U in City 1 and City 2, respectively. For individuals in states A and R, a reduction factor *γ* is introduced to quantify the protective effect induced by information, and the same protective effect is assumed for these two informed states, such that

(1) $$\beta_{1}^{A}=\beta_{1}^{R}=\gamma \beta_{1}^{U},\;\beta_{2}^{A}=\beta_{2}^{R}=\gamma \beta_{2}^{U},\;0\leq \gamma \leq 1.$$

A smaller γ indicates a stronger protective effect of information. When γ=1, information acquisition does not alter the infection risk, whereas γ=0 implies that susceptible individuals in states A and R are completely protected from infection. Since City 2 represents the larger city and is assumed to have a more intensive epidemic transmission environment, we set $\beta_{1}^{U}<\beta_{2}^{U}$. A proportional coefficient τ is further introduced to characterize the difference in disease transmission probabilities between the two cities:

(2) $$\beta_{1}^{X}=\tau \beta_{2}^{X},\;X\in \left\{U,A,R\right\},\;0<\tau <1.$$

Hereafter, we take $\beta =\beta_{2}^{U}$ as the reference disease transmission probability, so that $\beta_{1}^{U}=\tau \beta$. The information and disease spreading processes are coupled through the composite states of individuals, as illustrated in Figure 2f. According to their information and disease states, the model mainly contains five composite states: US, AS, RS, AI, and RI. Here, US, AS, and RS represent susceptible individuals in the unaware, active, and removed information states, respectively, whereas AI and RI denote infected individuals in the active and removed information states. The unaware–infected state UI may appear temporarily during a single-step update and in the subsequent probability-tree analysis, but it is not retained as a persistent state. Information states are updated before disease states, and infection probabilities depend on the updated information states. Newly infected individuals enter AI from U or A and RI from R. However, individuals in R at the start of the step end in RI if they become or remain infected, even if forgetting occurs during that step. Conversely, information diffusion affects disease spreading by reducing the infection risk of individuals in states A and R. Hence, information diffusion influences disease transmission, while infection can alter the information state of previously unaware individuals, resulting in a bidirectionally coupled spreading process.

## 3. Theoretical Analysis

In this section, we employ the MMC method to formulate the dynamical equations of the coupled information–epidemic spreading model with cross-city commuting and subsequently analyze its spreading behavior. Consider a two-city system consisting of City 1 and City 2 with population sizes $N_{1}$ and $N_{2}$, respectively, where the total population is $N=N_{1}+N_{2}$. Births, deaths, and permanent migration between the two cities are neglected. Let $A={\left(a_{ij}\right)}_{N\times N}$ denote the adjacency matrix of pairwise interactions in the information diffusion layer, where $a_{ij}=1$ if nodes *i* and *j* are connected and $a_{ij}=0$ otherwise. The 2-simplex structure in the information layer is represented by a third-order tensor $B={\left(b_{ijk}\right)}_{N\times N\times N}$, where $b_{ijk}=1$ if nodes *i*, *j*, and *k* jointly form a 2-simplex, and $b_{ijk}=0$ otherwise. For the disease spreading layer, $C\in {\left\{0,1\right\}}^{N_{1}\times N_{1}}$ and $D\in {\left\{0,1\right\}}^{N_{2}\times N_{2}}$ denote the intra-city contact networks of City 1 and City 2, respectively, while $F\in {\left\{0,1\right\}}^{N_{1}\times N_{2}}$ describes cross-city contacts generated when individuals from City 1 commute to City 2. Accordingly, the overall adjacency matrix of the disease spreading layer is given by

(3) $$G={\left(\begin{array}{cc} C & F\\ F^{T} & D \end{array}\right)}_{N\times N}.$$

Here, $F^{T}$ represents the same set of cross-city contacts from the perspective of nodes in City 2 and does not imply reverse commuting from City 2 to City 1. Since cross-city contacts arise only when individuals from City 1 commute to City 2 with commuting probability σ, the contribution of the contacts represented by **F** is modulated by σ in the subsequent transition probabilities. Let $p_{i}^{X}\left(t\right)$ denote the probability that node *i* is in composite state *X* at time *t*, where X∈{US,AS,RS,AI,RI}. Thus, for each node *i*, $p_{i}^{\mathrm{US}}\left(t\right)+p_{i}^{\mathrm{AS}}\left(t\right)+p_{i}^{\mathrm{RS}}\left(t\right)+p_{i}^{\mathrm{AI}}\left(t\right)+p_{i}^{\mathrm{RI}}\left(t\right)=1$. The state UI may appear as a transient intermediate state during a single-step update and in the probability-tree analysis, but it is not retained as a persistent composite state of the system.

To formulate the discrete-time evolution equations of individual states, we first define several probabilities associated with the information and disease spreading processes. The probabilities that node *j* is in the active information state and the infected state at time *t* are respectively given by

(4) $$p_{j}^{A}\left(t\right)=p_{j}^{\mathrm{AS}}\left(t\right)+p_{j}^{\mathrm{AI}}\left(t\right),\;p_{j}^{I}\left(t\right)=p_{j}^{\mathrm{AI}}\left(t\right)+p_{j}^{\mathrm{RI}}\left(t\right).$$

In the information diffusion layer, node *i* can acquire information through either pairwise interactions or higher-order interactions within 2-simplices. Let $r_{i}\left(t\right)$ denote the probability that node *i* does not receive information from its neighbors at time *t*. It is given by

(5) $$r_{i}\left(t\right)=\prod_{j=1}^{N}\left[1-\lambda a_{ij}p_{j}^{A}\left(t\right)\right]\prod_{\begin{array}{c} j<k\\ j,k\neq i \end{array}}\left[1-\lambda_{\Delta }b_{ijk}p_{j}^{A}\left(t\right)p_{k}^{A}\left(t\right)\right].$$

The first product represents the escape probability associated with pairwise information transmission, whereas the second accounts for higher-order transmission through 2-simplices. The higher-order transmission channel is activated only when the other two nodes in the same 2-simplex are simultaneously in state A.

For disease spreading, infection risk depends not only on an individual’s information state but also on intra-city contacts and unidirectional commuting from City 1 to City 2. To describe these infection channels in a unified form, we define the effective disease transmission matrix for information state X∈{U,A,R} as

(6) $$H^{X}=\left(\begin{array}{cc} \left(1-\sigma \right)\beta_{1}^{X}C\; & \sigma \beta_{2}^{X}F\\ \sigma \beta_{2}^{X}F^{T}\; & \beta_{2}^{X}D \end{array}\right),\;X\in \left\{U,A,R\right\},$$

where $h_{ij}^{X}$ denotes the (i,j)th element of $H^{X}$. The upper-left block describes local disease contacts in City 1 when individuals do not commute, the lower-right block represents local contacts within City 2, and the off-diagonal blocks characterize cross-city contacts generated by commuting from City 1 to City 2. Note that $F^{T}$ represents the same set of cross-city contacts from the perspective of nodes in City 2 and does not imply reverse commuting from City 2 to City 1. The model assumes that differences in effective transmission probabilities between cities reflect spatial heterogeneity in their local contact environments. During their stay in City 2, commuters from City 1 experience the transmission environment of City 2, so their contacts with local residents are governed by the destination city’s transmission conditions. Therefore, both cross-city contact blocks use $\beta_{2}^{X}$, covering transmission from commuters to local residents and in the opposite direction. In Equation (6), the potential within-city adjacency relations described by C and D remain fixed as σ varies, and the effect of commuting is incorporated through the prefactors of the corresponding matrix blocks. The factor 1−σ modulates the contribution of local contacts in City 1, whereas σ modulates the contribution of cross-city contacts. Consequently, increasing σ reduces the effective contribution of local contacts in City 1 and increases that of cross-city contacts, while preserving the within-city network topologies. Since residents of City 2 do not commute to City 1, their within-city contact block $\beta_{2}^{X}D$ is not directly modulated by σ.

Let $q_{i}^{X}\left(t\right)$ denote the probability that node *i*, when in information state *X*, escapes infection from all infected neighbors at time *t*. Then,

(7) $$q_{i}^{X}\left(t\right)=\prod_{j=1}^{N}\left[1-h_{ij}^{X}p_{j}^{I}\left(t\right)\right],\;X\in \left\{U,A,R\right\}.$$

Accordingly, $1-q_{i}^{X}\left(t\right)$ gives the corresponding infection probability. Based on the information-induced protection assumption

(8) $$\beta_{l}^{A}=\beta_{l}^{R}=\gamma \beta_{l}^{U},\;l\in \left\{1,2\right\},$$

we have

(9) $$H^{A}=H^{R}=\gamma H^{U}.$$

Nevertheless, $q_{i}^{A}\left(t\right)$ and $q_{i}^{R}\left(t\right)$ are retained separately in the following derivation to clearly distinguish the transitions among different composite states.

**Figure 3:**
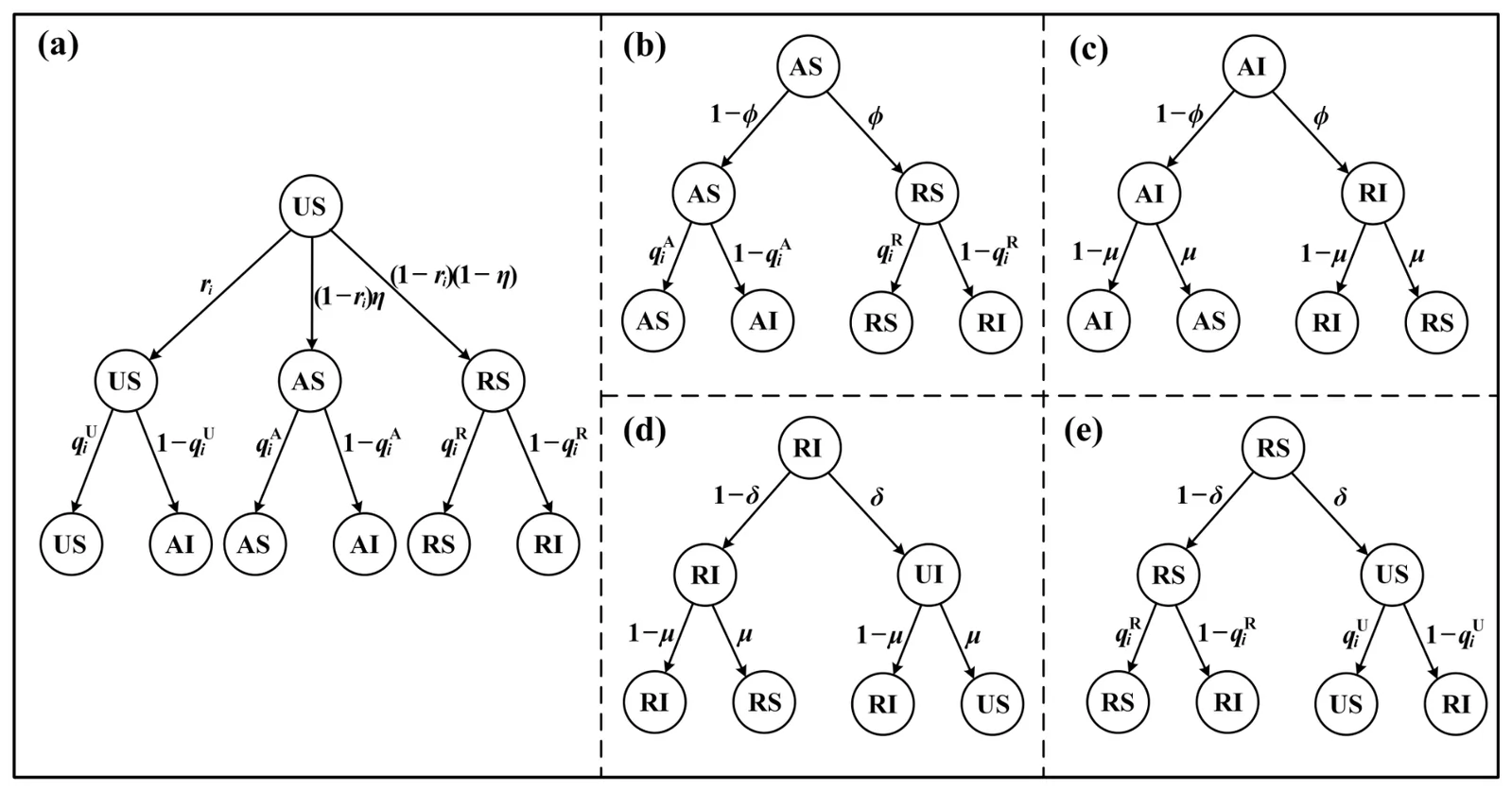
Probability trees for the transitions of the five composite states in the coupled information–epidemic spreading model. (**a**) Transitions from US; (**b**) transitions from AS; (**c**) transitions from AI; (**d**) transitions from RI; and (**e**) transitions from RS.

Based on the probabilities defined above, the possible transitions among the five composite states within one discrete time step are illustrated in Figure 3. A sequential updating scheme is adopted, in which the information state is updated first, followed by the disease state according to the updated information state. For individuals initially in state R, disease infection is assumed not to reactivate information dissemination. According to all possible transition paths shown in Figure 3a–e, the coupled spreading dynamics of node *i* can be formulated as follows:

(10) $$\left\{\begin{array}{cc} p_{i}^{\mathrm{US}}\left(t+1\right)= & r_{i}\left(t\right)q_{i}^{U}\left(t\right)p_{i}^{\mathrm{US}}\left(t\right)+\delta \mu p_{i}^{\mathrm{RI}}\left(t\right)+\delta q_{i}^{U}\left(t\right)p_{i}^{\mathrm{RS}}\left(t\right),\\ p_{i}^{\mathrm{AS}}\left(t+1\right)= & \eta \left[1-r_{i}\left(t\right)\right]q_{i}^{A}\left(t\right)p_{i}^{\mathrm{US}}\left(t\right)+\left(1-\phi \right)q_{i}^{A}\left(t\right)p_{i}^{\mathrm{AS}}\left(t\right)+\left(1-\phi \right)\mu p_{i}^{\mathrm{AI}}\left(t\right),\\ p_{i}^{\mathrm{AI}}\left(t+1\right)= & \left\{r_{i}\left(t\right)\left[1-q_{i}^{U}\left(t\right)\right]+\eta \left[1-r_{i}\left(t\right)\right]\left[1-q_{i}^{A}\left(t\right)\right]\right\}p_{i}^{\mathrm{US}}\left(t\right)\\ \; & +\left(1-\phi \right)\left[1-q_{i}^{A}\left(t\right)\right]p_{i}^{\mathrm{AS}}\left(t\right)+\left(1-\phi \right)\left(1-\mu \right)p_{i}^{\mathrm{AI}}\left(t\right),\\ p_{i}^{\mathrm{RS}}\left(t+1\right)= & \left(1-\eta \right)\left[1-r_{i}\left(t\right)\right]q_{i}^{R}\left(t\right)p_{i}^{\mathrm{US}}\left(t\right)+\phi q_{i}^{R}\left(t\right)p_{i}^{\mathrm{AS}}\left(t\right)+\phi \mu p_{i}^{\mathrm{AI}}\left(t\right)\\ \; & +\left(1-\delta \right)\mu p_{i}^{\mathrm{RI}}\left(t\right)+\left(1-\delta \right)q_{i}^{R}\left(t\right)p_{i}^{\mathrm{RS}}\left(t\right),\\ p_{i}^{\mathrm{RI}}\left(t+1\right)= & \left(1-\eta \right)\left[1-r_{i}\left(t\right)\right]\left[1-q_{i}^{R}\left(t\right)\right]p_{i}^{\mathrm{US}}\left(t\right)+\phi \left[1-q_{i}^{R}\left(t\right)\right]p_{i}^{\mathrm{AS}}\left(t\right)\\ \; & +\phi \left(1-\mu \right)p_{i}^{\mathrm{AI}}\left(t\right)+\left(1-\mu \right)p_{i}^{\mathrm{RI}}\left(t\right)\\ \; & +\left\{\left(1-\delta \right)\left[1-q_{i}^{R}\left(t\right)\right]+\delta \left[1-q_{i}^{U}\left(t\right)\right]\right\}p_{i}^{\mathrm{RS}}\left(t\right). \end{array}\right.$$

These equations are obtained by summing all possible transition paths leading to the corresponding composite state at time *t* + 1. The within-step transitions involving UI are incorporated into the corresponding terms of Equation (10). Specifically, in the update equation for $p_{i}^{\mathrm{AI}}\left(t+1\right)$, the term $r_{i}\left(t\right)\left[1-q_{i}^{U}\left(t\right)\right]p_{i}^{\mathrm{US}}\left(t\right)$ accounts for nodes initially in US that remain unaware after the information update and subsequently become infected. Under the assumed infection-induced awareness rule, these nodes pass through the transient state UI and enter AI with probability one within the same time step, so the probability contribution of this process is included directly in this term. Similarly, in the update equation for $p_{i}^{\mathrm{RI}}\left(t+1\right)$, the term $\delta \left[1-q_{i}^{U}\left(t\right)\right]p_{i}^{\mathrm{RS}}\left(t\right)$ accounts for nodes initially in RS that forget the information and subsequently become infected. According to the rule that infection does not reactivate information dissemination in individuals initially in R, these nodes end in RI, and their probability contribution is therefore included in the update equation for RI. The composite-state probabilities satisfy the normalization condition

(11) $$\sum_{X\in \{\mathrm{US},\mathrm{AS},\mathrm{AI},\mathrm{RS},\mathrm{RI}\}}p_{i}^{X}\left(t+1\right)=1.$$

To derive the epidemic threshold, we first define the total infection probability of node *i* as

(12) $$p_{i}^{I}\left(t\right)=p_{i}^{\mathrm{AI}}\left(t\right)+p_{i}^{\mathrm{RI}}\left(t\right).$$

By summing the evolution equations for $p_{i}^{\mathrm{AI}}\left(t+1\right)$ and $p_{i}^{\mathrm{RI}}\left(t+1\right)$, we obtain

(13) $$\begin{array}{cc} p_{i}^{I}\left(t+1\right)= & \left(1-\mu \right)p_{i}^{I}\left(t\right)\\ \; & +p_{i}^{\mathrm{US}}\left(t\right)\{r_{i}\left(t\right)\left[1-q_{i}^{U}\left(t\right)\right]+\left[1-r_{i}\left(t\right)\right][\eta \left[1-q_{i}^{A}\left(t\right)\right]\\ \; & \;\;\;+\left(1-\eta \right)[1-q_{i}^{R}\left(t\right)]]\}\\ \; & +p_{i}^{\mathrm{AS}}\left(t\right)\left\{\left(1-\phi \right)\left[1-q_{i}^{A}\left(t\right)\right]+\phi \left[1-q_{i}^{R}\left(t\right)\right]\right\}\\ \; & +p_{i}^{\mathrm{RS}}\left(t\right)\left\{\left(1-\delta \right)\left[1-q_{i}^{R}\left(t\right)\right]+\delta \left[1-q_{i}^{U}\left(t\right)\right]\right\}. \end{array}$$

At the disease-free equilibrium, no infected individuals are present, and hence

(14) $$p_{i}^{\mathrm{AI}*}=p_{i}^{\mathrm{RI}*}=p_{i}^{I*}=0.$$

Let ${\bar{p}}_{i}^{U}$, ${\bar{p}}_{i}^{A}$, and ${\bar{p}}_{i}^{R}$ denote the stationary probabilities of the three information states at the disease-free equilibrium, respectively. Thus,

(15) $${\bar{p}}_{i}^{U}+{\bar{p}}_{i}^{A}+{\bar{p}}_{i}^{R}=1.$$

The corresponding information escape probability is

(16) $$r_{i}^{*}=\prod_{j=1}^{N}\left[1-\lambda a_{ij}{\bar{p}}_{j}^{A}\right]\prod_{\begin{array}{c} j<k\\ j,k\neq i \end{array}}\left[1-\lambda_{\Delta }b_{ijk}{\bar{p}}_{j}^{A}{\bar{p}}_{k}^{A}\right].$$

At the disease-free equilibrium, the information dynamics satisfy

(17) $${\bar{p}}_{i}^{U}=r_{i}^{*}{\bar{p}}_{i}^{U}+\delta {\bar{p}}_{i}^{R}.$$

To examine the local stability of the disease-free equilibrium, we introduce a small infection perturbation in its neighborhood and let

(18) $$p_{i}^{I}\left(t\right)=p_{i}^{\mathrm{AI}}\left(t\right)+p_{i}^{\mathrm{RI}}\left(t\right)=\epsilon_{i}\left(t\right),\;\epsilon_{i}\left(t\right)\ll 1.$$

Accordingly, the infection escape probability can be expanded to first order as

(19) $$\begin{array}{cc} q_{i}^{X}\left(t\right) & =\prod_{j=1}^{N}\left[1-h_{ij}^{X}\epsilon_{j}\left(t\right)\right]\\ \; & ≃1-\sum_{j=1}^{N}h_{ij}^{X}\epsilon_{j}\left(t\right),\;X\in \left\{U,A,R\right\}, \end{array}$$

and hence

(20) $$1-q_{i}^{X}\left(t\right)≃\sum_{j=1}^{N}h_{ij}^{X}\epsilon_{j}\left(t\right).$$

Since

(21) $$H^{A}=H^{R}=\gamma H^{U},$$

substituting the above first-order approximation into the infection dynamics and neglecting higher-order terms yields

(22) $$\epsilon_{i}\left(t+1\right)≃\left(1-\mu \right)\epsilon_{i}\left(t\right)+\theta_{i}\sum_{j=1}^{N}h_{ij}^{U}\epsilon_{j}\left(t\right),$$

where

(23) $$\begin{array}{cc} \theta_{i}= & {\bar{p}}_{i}^{U}\left[r_{i}^{*}+\gamma \left(1-r_{i}^{*}\right)\right]+\gamma {\bar{p}}_{i}^{A}\\ \; & +\left[\delta +\gamma (1-\delta )\right]{\bar{p}}_{i}^{R}. \end{array}$$

Using

(24) $$r_{i}^{*}{\bar{p}}_{i}^{U}+\delta {\bar{p}}_{i}^{R}={\bar{p}}_{i}^{U}$$

together with the normalization condition, $\theta_{i}$ can be simplified to

(25) $$\theta_{i}={\bar{p}}_{i}^{U}+\gamma \left({\bar{p}}_{i}^{A}+{\bar{p}}_{i}^{R}\right)=\gamma +\left(1-\gamma \right){\bar{p}}_{i}^{U}.$$

Taking $\beta =\beta_{2}^{U}$ as the reference disease transmission probability and using

(26) $$\beta_{1}^{U}=\tau \beta_{2}^{U},$$

the effective transmission matrix can be expressed as

(27) $$H^{U}=\beta M,$$

where

(28) $$M=\left(\begin{array}{cc} (1-\sigma )\tau C\; & \sigma F\\ \sigma F^{T}\; & D \end{array}\right).$$

Define

(29) $$\Theta =\mathrm{diag}\left(\theta_{1},\theta_{2},\ldots ,\theta_{N}\right).$$

The linearized infection dynamics can then be written in matrix form as

(30) $$\epsilon \left(t+1\right)=\left[(1-\mu )I+\beta \Theta M\right]\epsilon \left(t\right),$$

where $\epsilon \left(t\right)={\left(\epsilon_{1}\left(t\right),\ldots ,\epsilon_{N}\left(t\right)\right)}^{T}$ and I is the N×N identity matrix. At a disease-free information equilibrium, small infection perturbations decay if

(31) $$\rho \;\left[(1-\mu )I+\beta \Theta M\right]<1,$$

where ρ(·) denotes the spectral radius of a matrix. If this information equilibrium is itself locally asymptotically stable, the above condition also implies local stability of the full disease-free equilibrium. Since ΘM is nonnegative, the boundary of the infection stability condition satisfies

(32) $$1-\mu +\beta_{c}\rho \left(\Theta M\right)=1.$$

For ρ(ΘM)>0, the critical transmission probability for local epidemic invasion around this information equilibrium is therefore

(33) $$\beta_{c}=\frac{\mu }{\rho (\Theta M)}.$$

## 4. Simulation Results

In the numerical simulations, both the MMC method and MC simulations are employed to investigate the stationary spreading behavior of the system. Unless otherwise specified, the upper information layer is constructed as a Barabási–Albert (BA) network with 2-simplices, containing N=2000 nodes, with mean degree 10 and mean 2-simplex degree 2. Specifically, a pairwise network is first generated according to the BA mechanism, after which explicit 2-simplices are randomly introduced according to the prescribed mean 2-simplex degree. The three pairwise edges associated with each generated 2-simplex are incorporated into the network, resulting in an information layer containing both pairwise and higher-order interactions. When randomly adding 2-simplices, different simplices are allowed to share nodes or edges, and overlapping simplices are retained as distinct higher-order interaction units. The same set of three nodes defines a single 2-simplex regardless of node ordering and is recorded only once. The three pairwise edges of each generated 2-simplex are included in the pairwise network: existing connections are retained, while missing connections are added. An edge shared by multiple 2-simplices is represented by a single link in the pairwise network and is not counted repeatedly because of its participation in multiple simplices. Only explicitly selected triples are designated as 2-simplices; other triangular cliques formed by pairwise connections are not automatically assigned higher-order interactions. The lower disease layer consists of City 1 and City 2 with $N_{1}=500$ and $N_{2}=1500$ nodes, respectively. The mean degree of both intra-city networks is set to 6, and Erdős–Rényi (ER) and BA structures are considered. The number of potential contacts of each City-1 individual in City 2 ranges from 1 to 6 and follows a truncated Poisson distribution with a mean of approximately 3. To characterize the spreading states, $\rho^{A}$ and $\rho^{R}$ denote the densities of individuals in the active and removed information states, respectively, while $\rho^{I}$ denotes the density of infected individuals. In the MMC calculations, $p_{i}^{\mathrm{AI}}\left(0\right)=0.01$ and $p_{i}^{\mathrm{US}}\left(0\right)=0.99$, with all other state probabilities initially set to zero. In the MC simulations, 1% of the nodes are randomly initialized in state AI, while the remaining nodes are initialized in state US. For the MMC calculations, convergence was checked from the 300th time step onward. Convergence was declared when the maximum absolute change across all node-level composite-state probabilities between successive time steps remained below $10^{-9}$ for 20 consecutive steps. The whole-system and city-specific infection densities were obtained by averaging the node infection probabilities at the final iteration over the corresponding node populations. For the MC simulations, each realization was run for 3000 time steps, with the first 2900 steps treated as a relaxation period and the infection densities averaged over the final 100 steps. These time averages were then averaged over 50 independent stochastic realizations on the same network. The instantaneous infection densities of the whole system and the two cities were calculated by dividing the corresponding numbers of infected nodes by the population sizes *N*, $N_{1}$, and $N_{2}$, respectively.

**Figure 4:**
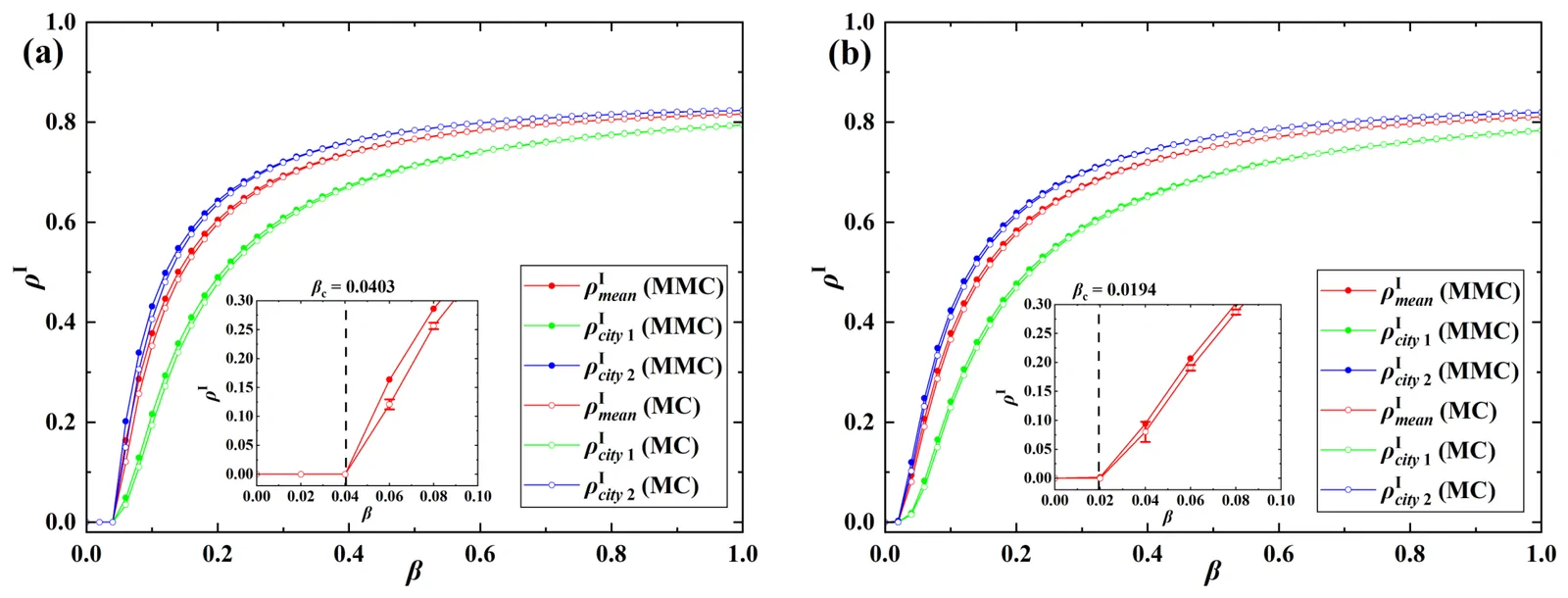
Comparison between the MMC and MC results for the steady-state infection densities as a function of *β*. (**a**) ER networks for both cities in the lower disease layer; (**b**) BA networks for both cities in the lower disease layer. $\rho_{\mathrm{mean}}^{I}$, $\rho_{\mathrm{city}\;1}^{I}$, and $\rho_{\mathrm{city}\;2}^{I}$ denote the infection densities of the whole system, City 1, and City 2, respectively. Filled and open symbols represent the MMC and MC results, respectively. The MC results are averaged over 50 independent realizations. The insets show the whole-system infection density near the threshold. Vertical dashed lines mark the theoretical epidemic thresholds calculated from Equation (33) for the corresponding networks and parameter settings. Error bars at selected MC points in the insets represent the sample standard deviation of the time-averaged infection densities across the 50 independent realizations. The parameters are λ=0.08, $\lambda_{\Delta }=0.2$, ϕ=0.2, δ=0.1, η=0.8, μ=0.2, γ=0.5, τ=0.6, and σ=0.2, with $\beta =\beta_{2}^{U}$ and $\beta_{1}^{U}=\tau \beta$.

We first assess the accuracy of the MMC predictions by comparing them with MC simulations. Figure 4 shows that, for both lower-layer network configurations, the MMC predictions and MC simulations exhibit consistent overall trends in the stationary infection densities of the whole system and the two cities as β varies. Filled and open symbols represent the MMC and MC results, respectively. The insets further resolve the whole-system infection density near the threshold. The theoretical thresholds calculated from Equation (33) are approximately $\beta_{c}=0.0403$ for the ER lower-layer networks and $\beta_{c}=0.0194$ for the BA lower-layer networks. At the resolution of the present β scan, these theoretical thresholds are broadly consistent with the onset region observed in the MC simulations. Minor deviations between the MMC and MC results occur near the threshold, with closer agreement at larger values of β. As β increases, all three infection densities remain close to zero at small β, increase rapidly after the epidemic threshold is exceeded, and then gradually approach saturation. A comparison between Figure 4a,b shows that the lower-layer network structure has a clear influence on the epidemic threshold. When the intra-city networks of both cities are BA networks, the infection densities begin to increase at a smaller value of β, indicating a lower epidemic threshold than that observed for the ER networks. From the discrete values shown in the figure, a noticeable nonzero prevalence already appears around β=0.04 for the BA case, whereas the infection density remains close to zero for the ER case at the same value. This suggests that the heterogeneous connectivity of BA networks facilitates epidemic persistence at lower transmission probabilities, which is consistent with the role of highly connected nodes in promoting disease transmission. Beyond the threshold, both network configurations exhibit $\rho_{\mathrm{city}\;2}^{I}>\rho_{\mathrm{mean}}^{I}>\rho_{\mathrm{city}\;1}^{I}$. This behavior results from $\beta_{1}^{U}=\tau \beta_{2}^{U}$ with τ<1, leading to a higher infection level in City 2. Moreover, because City 2 contains a larger fraction of the population, the overall infection density is closer to that of City 2. As β further increases, the differences in prevalence between the two network structures gradually decrease, indicating that the influence of network topology is more pronounced near the epidemic threshold.

**Figure 5:**
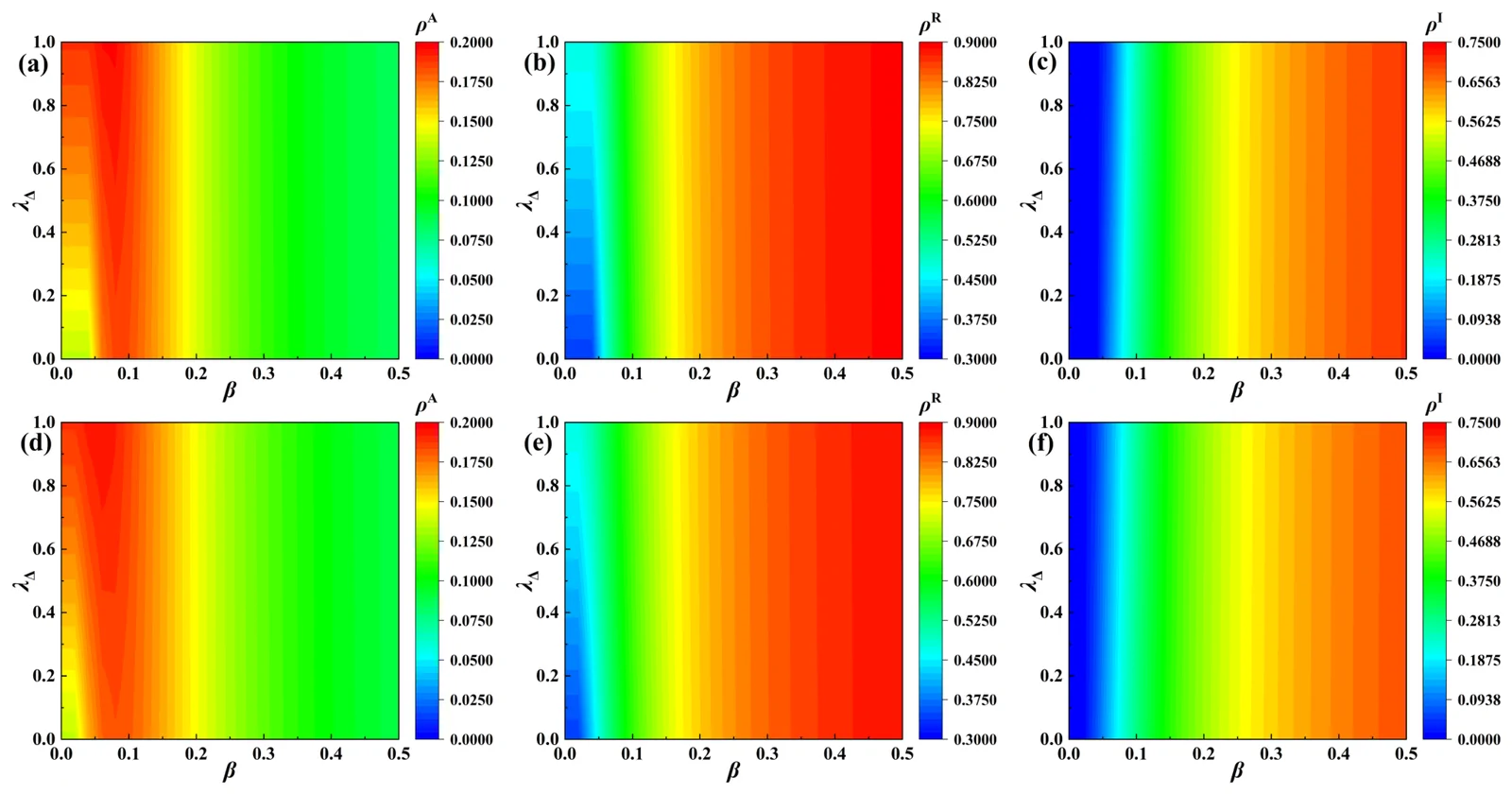
Steady-state densities $\rho^{A}$, $\rho^{R}$, and $\rho^{I}$ as functions of the basic disease transmission probability β and the higher-order information transmission probability $\lambda_{\Delta }$. Panels (**a**–**c**) correspond to ER networks for both cities in the lower disease layer, whereas panels (**d**–**f**) correspond to BA networks for both cities. Panels (**a**,**d**) show $\rho^{A}$, panels (**b**,**e**) show $\rho^{R}$, and panels (**c**,**f**) show $\rho^{I}$. The parameters are λ=0.05, η=0.8, ϕ=0.2, δ=0.1, μ=0.2, γ=0.3, τ=0.6, and σ=0.2.

Following this comparison, we examine how information transmission through group interactions influences the coupled spreading process. Figure 5 illustrates the joint effects of the basic disease transmission probability β and the higher-order information transmission probability $\lambda_{\Delta }$ on the steady-state behavior of the system. As β increases, $\rho^{I}$ gradually rises from a value close to zero and eventually reaches a relatively high stationary infection level, while $\rho^{R}$ also increases markedly. In contrast, $\rho^{A}$ exhibits a pronounced nonmonotonic dependence on β. In the small-β region, the increase in infection prevalence causes more individuals initially in the U state to become active information spreaders through infection-induced information acquisition, thereby increasing $\rho^{A}$. As β further increases, the sustained high infection level continues to promote information acquisition; meanwhile, more individuals in the A state gradually accumulate in the R state through the transition from the A state to the R state. Consequently, $\rho^{R}$ continues to increase, whereas $\rho^{A}$ decreases after reaching its maximum. Therefore, the peak of $\rho^{A}$ reflects the competition between infection-induced information activation and the transition of active individuals to the removed state, while the continuous increase in $\rho^{R}$ indicates that an increasing number of individuals experience information acquisition followed by withdrawal from information spreading.

The effect of higher-order information transmission on infection density is closely related to changes in the combined informed proportion, $\rho^{A}+\rho^{R}=1-\rho^{U}$. In the small-β region, increasing $\lambda_{\Delta }$ can raise both $\rho^{A}$ and $\rho^{R}$, primarily reflecting an expansion of the informed population. For example, at β=0.06, increasing $\lambda_{\Delta }$ from 0 to 1 raises the combined informed proportion from approximately 0.6516 to 0.7138 in the ER lower-layer networks and from approximately 0.6918 to 0.7549 in the BA lower-layer networks. Individuals in state A participate in information dissemination, whereas those in state R have withdrawn from it. According to Equation (1), susceptible individuals in both states have the same protection factor γ. Thus, the additional direct protection in this region primarily arises when previously unaware susceptible individuals acquire information, whereas redistribution between the two informed states does not itself strengthen individual protection. As β increases, infection-induced information acquisition sustains a high informed proportion, and the additional information coverage provided by stronger higher-order transmission becomes progressively smaller. For example, at β=0.30, the same variation in $\lambda_{\Delta }$ raises the combined informed proportion only from approximately 0.9598 to 0.9605 in the ER networks and from approximately 0.9552 to 0.9561 in the BA networks, with both increases remaining below 0.1 percentage points. These results indicate that, under the present parameter settings, higher-order transmission can appreciably suppress infection near the threshold by expanding the informed population, while the weaker response of infection density at larger β is primarily associated with limited additional protective coverage. A comparison between the upper and lower rows further shows that the overall evolution patterns of the three state densities are similar for ER and BA lower-layer networks, although differences occur in the locations of the transition regions and in the stationary levels. In particular, the low-infection region in the BA case contracts toward smaller values of β, indicating that degree heterogeneity allows the disease to persist at lower transmission probabilities. Meanwhile, the resulting change in epidemic spreading conditions feeds back to the information layer through the information–epidemic coupling mechanism, leading to quantitative differences in $\rho^{A}$ and $\rho^{R}$ between the two network structures.

**Figure 6:**
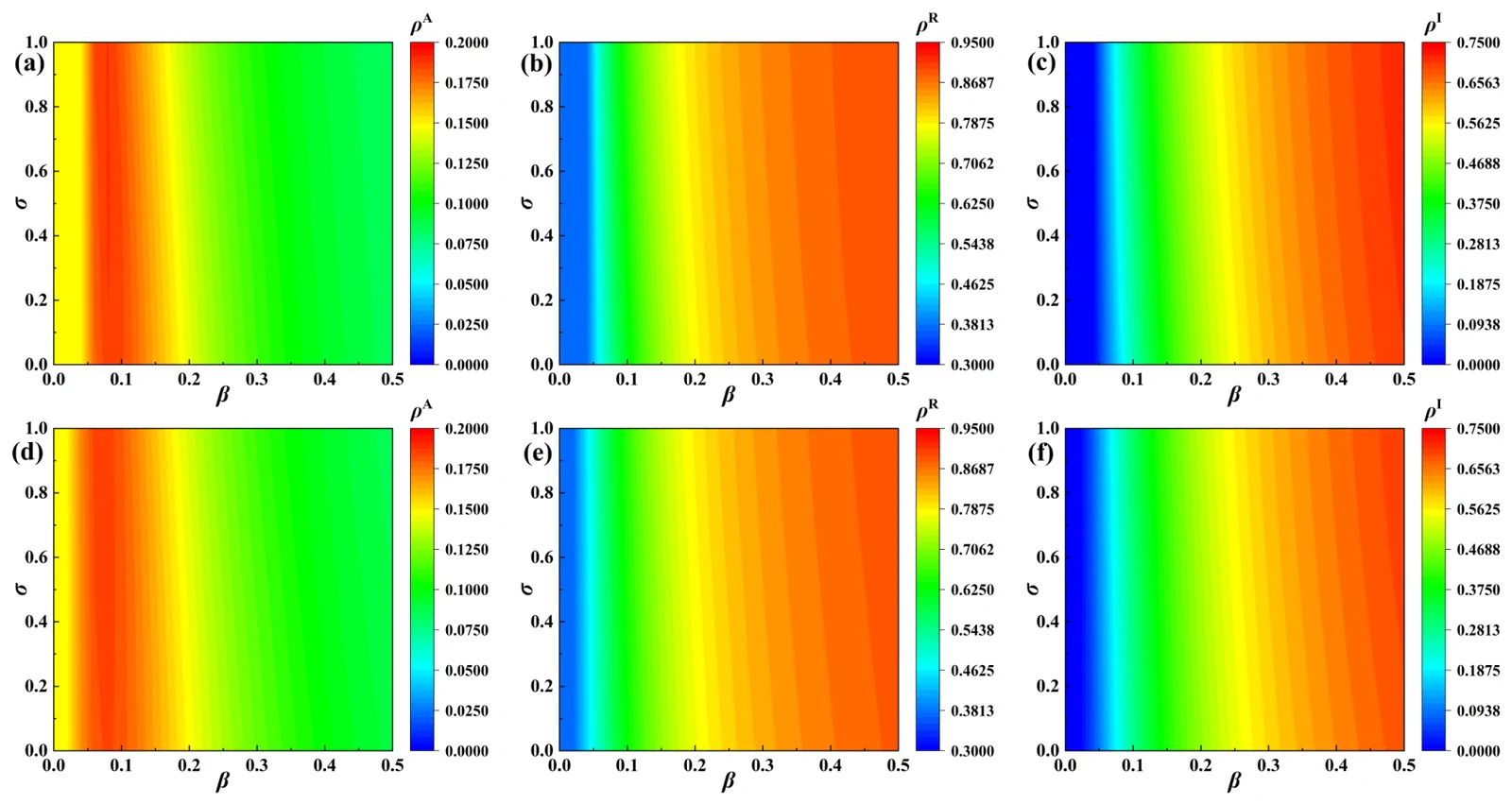
Steady-state densities $\rho^{A}$, $\rho^{R}$, and $\rho^{I}$ as functions of the basic disease transmission probability β and the commuting probability σ. Panels (**a**–**c**) correspond to ER networks for both cities in the lower disease layer, whereas panels (**d**–**f**) correspond to BA networks for both cities. Panels (**a**,**d**) show $\rho^{A}$, panels (**b**,**e**) show $\rho^{R}$, and panels (**c**,**f**) show $\rho^{I}$. The parameters are λ=0.05, $\lambda_{\Delta }=0.2$, η=0.8, ϕ=0.2, δ=0.1, μ=0.2, γ=0.3, and τ=0.6.

We now turn to intercity commuting to examine how changes in physical contacts affect epidemic spreading and the associated information dynamics. Figure 6 illustrates the joint effects of the basic disease transmission probability β and the commuting probability σ on the steady-state spreading behavior of the system. Overall, β remains the dominant factor governing the three state densities. As β increases, both $\rho^{I}$ and $\rho^{R}$ increase markedly, whereas $\rho^{A}$ first increases and then gradually decreases, exhibiting a nonmonotonic pattern similar to that observed in Figure 5. For a given β, increasing σ further changes the stationary distribution of the three states. In particular, $\rho^{I}$ generally increases with stronger commuting, accompanied by a corresponding increase in $\rho^{R}$. Since the commuting probability directly regulates cross-city epidemic contacts, its variation first affects the epidemic process and then feeds back to the information layer through the information–epidemic coupling. Therefore, the responses of $\rho^{A}$ and $\rho^{R}$ to σ can be regarded as indirect consequences of changes in epidemic spreading. Compared with β, the effect of σ on the stationary densities is relatively moderate, but it is more evident in the small- and intermediate-β regions.

Figure 6c,f further show that commuting affects not only the epidemic prevalence after an outbreak but also the condition for epidemic persistence. As σ increases, the transition boundary between the low-infection and persistent-spreading regions shifts toward smaller values of β, indicating that stronger cross-city commuting enables the epidemic to persist at a lower basic transmission probability. A comparison between the upper and lower rows shows that the overall effects of β and σ are qualitatively similar for ER and BA lower-layer networks, suggesting that the influence of commuting is not restricted to a particular intra-city topology. Nevertheless, clear quantitative differences remain in the transition regions and stationary densities. Compared with the ER case, the persistent-infection region in the BA networks emerges at smaller values of β, indicating that degree heterogeneity further reduces the transmission conditions required for epidemic persistence. These results show that intra-city network topology and cross-city commuting jointly determine the epidemic outbreak condition and stationary prevalence, while their effects are further transmitted to the information layer through the coupling mechanism.

**Figure 7:**
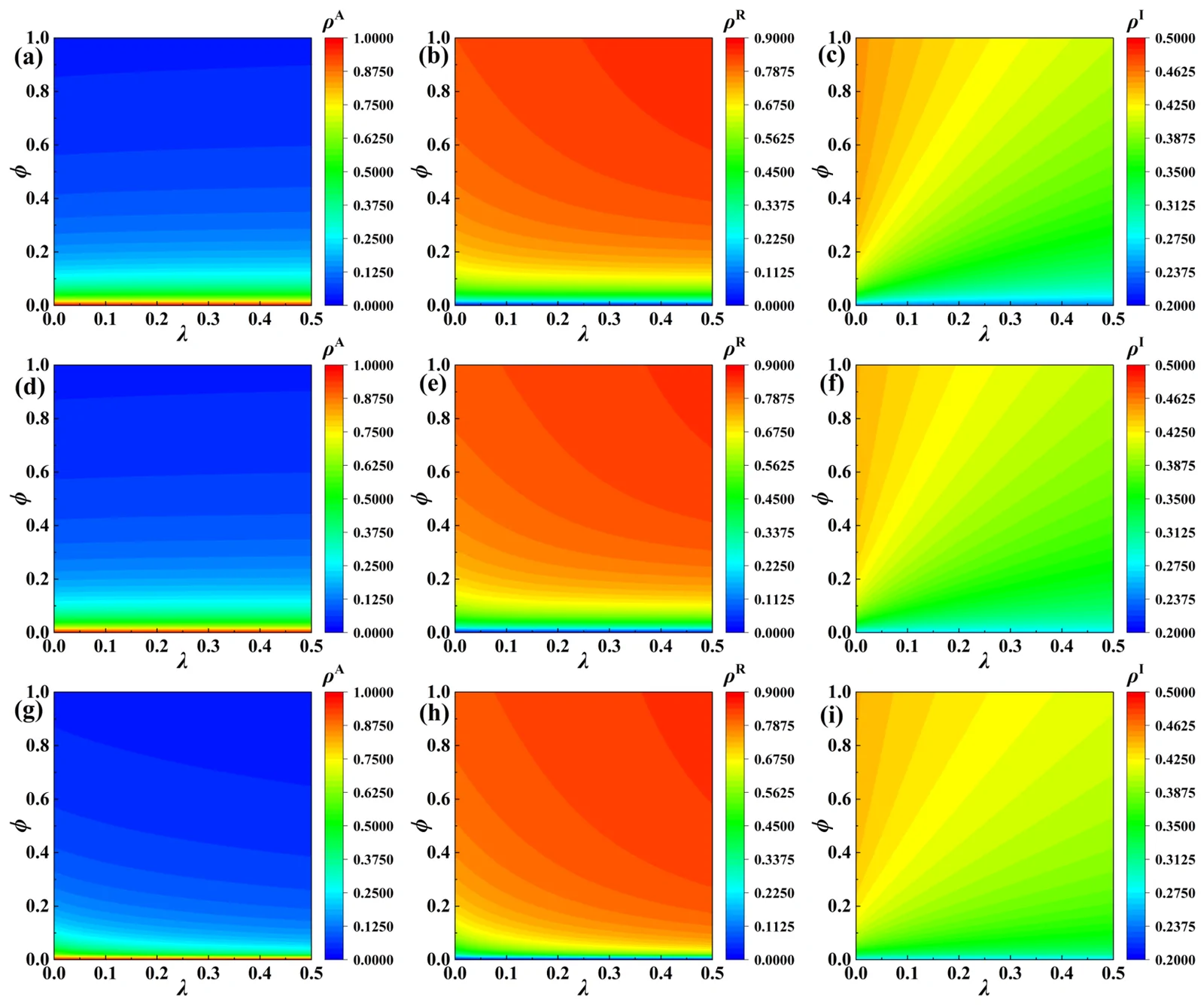
Steady-state densities $\rho^{A}$, $\rho^{R}$, and $\rho^{I}$ as functions of the pairwise information transmission probability λ and the transition probability ϕ. Panels (**a**–**c**) correspond to ER networks for both cities in the lower disease layer with η=0.8; panels (**d**–**f**) correspond to BA networks for both cities with η=0.8; and panels (**g**–**i**) correspond to the same BA network configuration as panels (**d**–**f**), but with η=0.2. Panels (**a**,**d**,**g**) show $\rho^{A}$; panels (**b**,**e**,**h**) show $\rho^{R}$; and panels (**c**,**f**,**i**) show $\rho^{I}$. The other parameters are $\lambda_{\Delta }=0.2$, δ=0.1, β=0.15, μ=0.2, γ=0.3, τ=0.6, and σ=0.2.

The different responses of the active and removed populations motivate a closer examination of information acquisition and withdrawal from active spreading. Figure 7 illustrates the joint effects of the pairwise information transmission probability λ and the transition probability ϕ on the information states and epidemic spreading. Overall, ϕ has a pronounced influence on the composition of the information states. As ϕ increases, $\rho^{A}$ decreases markedly, whereas $\rho^{R}$ continuously increases, because a larger ϕ accelerates the departure of active individuals from the A state and their transition to the R state. Meanwhile, λ changes the information-state distribution by promoting pairwise information transmission and further feeds back to the epidemic process. Figure 7c,f,i show that, for a given ϕ, increasing λ generally reduces $\rho^{I}$, whereas a larger ϕ tends to increase the infection level. Although individuals in the A and R states have the same protection factor against infection, only those in the A state can continue to disseminate information. Therefore, a larger ϕ weakens sustained information diffusion and indirectly reduces its suppressive effect on epidemic spreading. These results indicate that λ and ϕ jointly regulate the coupled information–epidemic dynamics by promoting information acquisition and controlling the persistence of active information spreaders, respectively.

A comparison between Figure 7a–f shows that, for η=0.8, the overall dependence on λ and ϕ is similar for ER and BA lower-layer networks, with only quantitative differences in the stationary densities. Since Figure 7d–i use the same BA lower-layer configuration, their differences mainly arise from reducing η from 0.8 to 0.2. Notably, the response of $\rho^{A}$ to λ differs between Figure 7d,g. When η is large, newly informed individuals are more likely to enter the A state and continue disseminating information; therefore, increasing λ can effectively replenish active spreaders, making the dependence of $\rho^{A}$ on λ relatively weak or jointly controlled by other state transitions. In contrast, when η=0.2, most newly informed individuals enter the R state, so increasing λ no longer efficiently replenishes the A population. At the same time, broader information diffusion suppresses infection and consequently reduces the number of AI individuals generated directly through infection. These two effects together lead to a different dependence of $\rho^{A}$ on λ in Figure 7g, with a more evident decreasing tendency than that in Figure 7d. The comparison between Figure 7f and i shows that η regulates stationary infection density through sustained information dissemination and informed coverage. According to Equation (1), susceptible individuals in states A and R have the same protection factor γ, so reducing η does not directly weaken the protection of informed individuals. However, decreasing η from 0.8 to 0.2 directs more newly informed individuals into state R, reducing the replenishment of active spreaders and limiting information provision to other individuals in state U. For example, at λ=ϕ=0.2, the increase in $\rho^{R}$ does not compensate for the larger decrease in $\rho^{A}$: the combined informed proportion decreases from approximately 0.9145 to 0.8975, while infection density increases from approximately 0.3724 to 0.3919. Thus, a larger population in state R does not necessarily imply improved population-level protection; sustained information dissemination must also maintain informed coverage. Meanwhile, at η=0.2, increasing λ at fixed ϕ can reduce $\rho^{A}$, while the larger increase in $\rho^{R}$ raises the combined informed proportion and infection density continues to decline. Equation (5) shows that the information acquisition probability $1-r_{i}\left(t\right)$ depends jointly on transmission probabilities and the probabilities of neighboring nodes being in state A. Hence, fewer active spreaders can coexist with stronger epidemic suppression. These results distinguish the direct protection of informed individuals from the indirect protection provided to others through sustained information dissemination.

**Figure 8:**
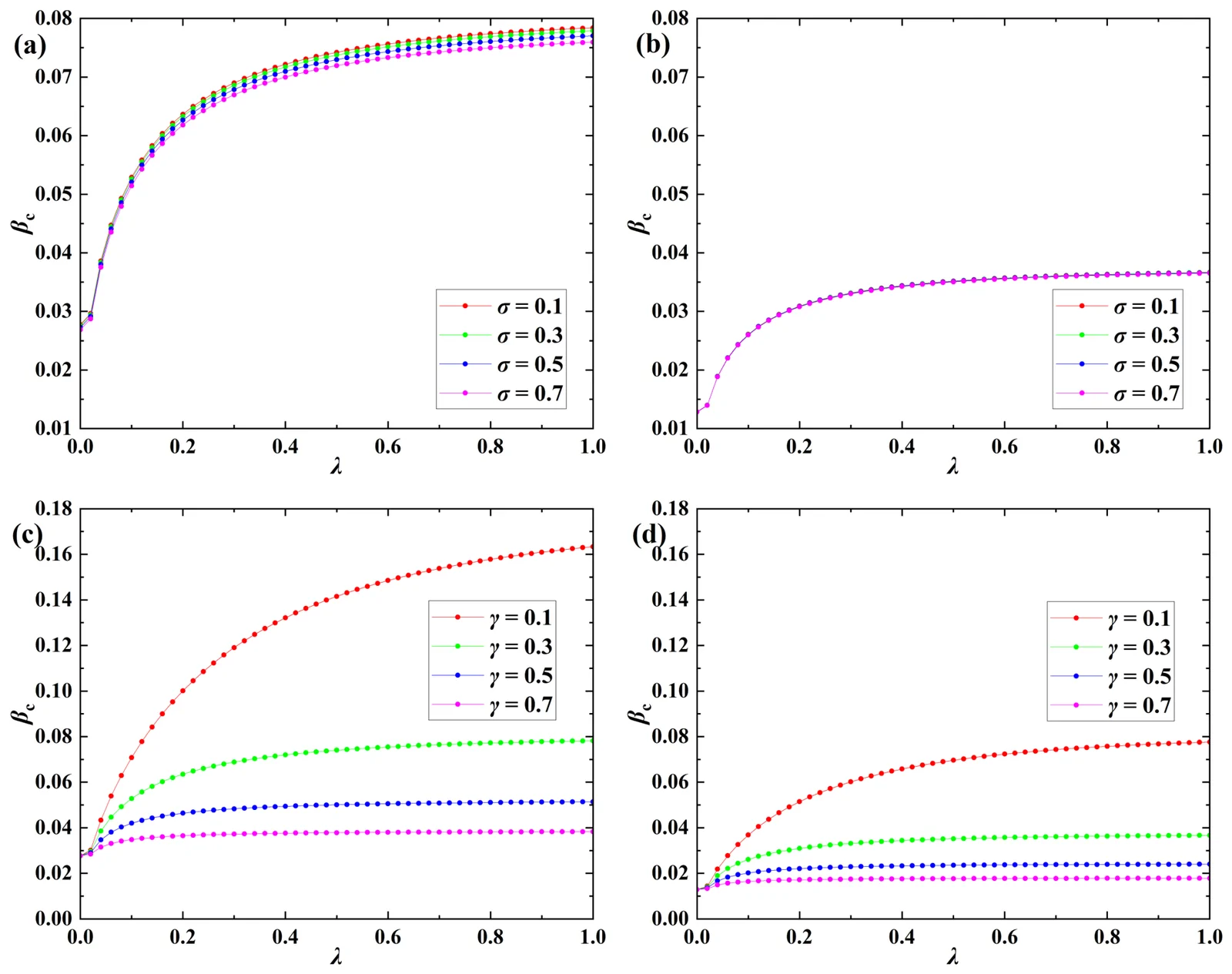
Epidemic threshold $\beta_{c}$ as a function of the pairwise information transmission probability λ. (**a**) ER networks for both cities in the lower disease layer with σ=0.1,0.3,0.5, and 0.7; (**b**) BA networks for both cities with the same values of σ. In (**a**,**b**), γ=0.3. (**c**) ER networks for both cities with γ=0.1,0.3,0.5, and 0.7; (**d**) BA networks for both cities with the same values of γ. In (**c**,**d**), σ=0.2. The other parameters are $\lambda_{\Delta }=0.2$, η=0.8, ϕ=0.2, δ=0.1, μ=0.2, and τ=0.6.

To complement the stationary-state analysis, we finally examine the conditions for local epidemic invasion using the derived critical transmission probability. Figure 8 illustrates the effect of the pairwise information transmission probability λ on the epidemic threshold $\beta_{c}$, together with the roles of the commuting probability σ and the protection factor γ under different lower-layer network structures. In all four panels, $\beta_{c}$ increases markedly with λ and gradually approaches a plateau at large λ, indicating that enhanced pairwise information transmission raises the transmission condition required for an epidemic outbreak, although this effect becomes progressively weaker as λ increases. Figure 8a,b further show that the influence of commuting depends on the lower-layer topology. For the ER networks, increasing σ slightly decreases $\beta_{c}$, and the separation among the curves becomes more visible as λ increases, indicating that stronger cross-city commuting can facilitate epidemic spreading to some extent. In contrast, the curves corresponding to different values of σ almost overlap in the BA networks, suggesting that the epidemic threshold is relatively insensitive to commuting under the present parameter settings. Moreover, a comparison between Figure 8a,b shows that the BA networks exhibit consistently lower values of $\beta_{c}$ than the ER networks, indicating that degree heterogeneity reduces the critical transmission probability required for sustained epidemic spreading.

This difference can be interpreted using the threshold relation $\beta_{c}=\mu /\rho \left(\Theta M\right)$ in Equation (33). At the same mean degree, BA networks exhibit stronger degree heterogeneity. Highly connected nodes and their neighborhoods provide multiple transmission paths that support local persistence through recurrent infection, contributing to a larger spectral radius of the effective transmission matrix and hence a lower epidemic threshold. According to Equation (28), varying σ modifies both the within-city contact contribution in City 1 and the cross-city contact contributions, whereas the within-city block D of City 2 is not directly modulated by σ. Thus, the within-city transmission support provided by highly connected nodes and their neighborhoods in City 2 persists as commuting varies. Under the present network and parameter settings, the nearly overlapping threshold curves for the BA networks are consistent with commuting-induced contact reweighting having a relatively small effect on the overall spectral radius compared with this strong within-city transmission support. In the ER networks, the degree distribution is more homogeneous and highly connected nodes play a less prominent role, so changes in cross-city contact weights have a more noticeable relative effect on effective transmission, consistent with the clearer threshold differences in Figure 8a. The observed difference in commuting sensitivity therefore reflects the joint effects of degree heterogeneity and the adopted asymmetric commuting structure.

Figure 8c,d show that the protection factor γ has a much stronger influence on the epidemic threshold. As γ increases from 0.1 to 0.7, $\beta_{c}$ decreases substantially, and the separation among the curves becomes increasingly pronounced with increasing λ. This indicates that when information spreads more extensively, the protective effect induced by information plays a more important role in determining the epidemic outbreak condition. A smaller γ corresponds to stronger protection and therefore leads to a markedly higher epidemic threshold, whereas at small λ, the limited spread of information restricts the differences caused by different protection levels. A comparison of the two parameters further shows that, within the present parameter ranges, γ has a considerably stronger effect on $\beta_{c}$ than σ. This behavior is consistent with the theoretical relation $\beta_{c}=\mu /\rho \left(\Theta M\right)$: λ and γ affect the threshold through the disease-free information distribution and the protection matrix Θ, whereas σ reweights both within-city contacts in City 1 and cross-city contacts in the effective transmission matrix M. Therefore, the epidemic outbreak condition is jointly determined by information spreading, information-induced protection, and the topology of the lower-layer city networks.

## 5. Conclusions

This study developed a two-layer model of coupled information–epidemic spreading incorporating higher-order information interactions and asymmetric intercity commuting. The model preserves within-city contact structures, introduces additional cross-city contacts generated by commuting, and accounts for differences in population size and transmission conditions. Using the MMC method, we formulated the evolution equations for node-state probabilities and derived the critical transmission probability $\beta_{c}$. The predicted stationary infection densities agreed well with MC simulations. The numerical results showed that stronger pairwise information transmission generally reduces infection density and increases $\beta_{c}$, although the latter effect gradually saturates. Higher-order information transmission mainly modifies the distribution of information states, with a comparatively limited effect on infection density under the present parameter settings. Stronger commuting generally increases stationary prevalence, whereas its influence on $\beta_{c}$ depends on the city network structure: increasing commuting intensity slightly reduces $\beta_{c}$ in ER networks but produces relatively small changes in BA networks. Moreover, BA networks consistently exhibit lower values of $\beta_{c}$ than ER networks. Within the investigated parameter ranges, variations in the protection factor produced larger changes in $\beta_{c}$ than variations in commuting intensity.

These findings highlight the joint roles of sustained information dissemination, information-induced protection, and city contact structures in intercity epidemic spreading. Even when active spreaders and informed individuals who no longer disseminate information have identical protection levels, faster withdrawal from active spreading can weaken sustained information diffusion and its collective suppressive effect. Maintaining awareness and continuing to disseminate information therefore have distinct implications for epidemic dynamics. The present model assumes static contact networks, fixed spreading parameters, and recurrent commuting initiated only by residents of the smaller city, and it has not yet been calibrated using empirical city data. Future work could extend the framework to multiple cities, bidirectional or time-varying commuting, and heterogeneous protective behaviors, while incorporating empirical mobility data to assess its applicability.

## Data Availability

All data that support the findings of this study are included within the article.
